# Updated distributional checklist of the genus *Pytho* Latreille, 1796 of the Palearctic realm with the first records of *P.abieticola* J. R. Sahlberg, 1875 from Lithuania and the family Pythidae (Coleoptera) from Moldova and Serbia

**DOI:** 10.3897/BDJ.12.e115422

**Published:** 2024-01-24

**Authors:** Radomir Jaskuła, Denis Ćoso, Vytautas Tamutis, Romas Ferenca

**Affiliations:** 1 Department of Invertebrate Zoology and Hydrobiology, Faculty of Biology and Environmental Protection, University of Lodz, Banacha 12/16, PL-90-237, Łódź, Poland Department of Invertebrate Zoology and Hydrobiology, Faculty of Biology and Environmental Protection, University of Lodz, Banacha 12/16, PL-90-237 Łódź Poland; 2 Timočke Divizije 6/6, Voždovac, Belgrade, Serbia Timočke Divizije 6/6, Voždovac Belgrade Serbia; 3 Kaunas Tadas Ivanauskas Museum of Zoology, Laisvės alėja str. 106, LT-44253, Kaunas, Lithuania Kaunas Tadas Ivanauskas Museum of Zoology, Laisvės alėja str. 106, LT-44253 Kaunas Lithuania; 4 Vytautas Magnus Universtity, K. Donelaičio str. 58, 44248, Kaunas, Lithuania Vytautas Magnus Universtity, K. Donelaičio str. 58, 44248 Kaunas Lithuania

**Keywords:** Pythidae, *Pytho*, dead log bark beetles, saproxylic beetles, species list, new records, Lithuania, Moldova, Serbia, citizen science, iNaturalist

## Abstract

**Background:**

*Pytho* Latreille, 1796 is a small genus of the dead log bark beetles (Coleoptera, Pythidae). All species are distributed in the Holarctic, being recognised as typically boreal taxa, but knowledge about the geographical ranges of particular taxa is far from complete.

**New information:**

The updated distributional checklist of the genus *Pytho* of the Palearctic is given, based on literature and new records, including citizen-scientific data. *Pythodepressus* and the family Pythidae are recorded for the first time from the Republic of Moldova (Municipality of Chișinău) and the Republic of Serbia (Municipality of Voždovac) and *P.abieticola* is recorded for the first time from Lithuania (Alytus District Municipality, Ignalina District Municipality and Kaišiadorys District Municipality).

## Introduction

The genus *Pytho* Latreille, 1796 is one of only seven genera classified in the small tenebrionid beetle family Pythidae Solier, 1834, the common name of which is 'dead log bark beetles' (Pollock 1991, Pollock 1998). Although with the occurrence in the Holarctic realm it has the widest distribution amongst all pythid genera, the knowledge about species ranges of particular taxa is far from complete. This may be due to their small body size (usually ca. 9-15 mm) and hidden lifestyle (larvae live under the bark of trees, adults are active mainly during the night). The genus includes 10 species of which four occur in the Nearctic and six are known from the Palaearctic (Pollock 1991, Pollock and Iwan 2020, Háva and Zahradník 2021). Most species are recognised as typically boreal taxa and cold boreal regions are characterised by highest *Pytho* species richness. Adults of this genus prefer large tree trunks of dead conifers (*Picea*, *Abies*, *Larix*), but occasionally can be found on deciduous trees (*Betula*, *Salix*, *Populus*), too, both standing and fallen trees as well as on tree stumps (e.g. Burakowski 1962, Pettersson 1983, Pollock 1991, Miessen 1994, Siitonen and Saaristo 2000, Smith and Sears 2012, Kapla et al. 2021, Chittaro et al. 2023). In Europe, only three species of *Pytho* have been noted: *P.abieticola* J. R. Sahlberg, 1875, *P.depressus* Linnaeus, 1767 and *P.kolwesis* C. R. Sahlberg, 1833. *P.depressus* is characterised by the largest species range amongst all Palaearctic *Pytho* species, covering great parts of Europe and Asia (Iablokoff-Khnzorian 1985, Egorov 1992, Kim et al. 2004, Pollock and Iwan 2020, Háva and Zahradník 2021, Kapla et al. 2021, Thomaes et al. 2022).

The aim of the paper is to present the first evidence of the occurrence of *P.depressus* and, thus, the family Pythidae from the Republic of Moldova and the Republic of Serbia, as well as the first distributional data for *P.abieticola* from Lithuania. In addition, an updated distributional checklist for genus *Pytho* from the Palaearctic realm is provided by adding recent literature records as well as data overlooked in both editions of the Catalogue of Palaearctic Coleoptera (Pollock 2008, Pollock and Iwan 2020) and the Fauna Europea database (Vazquez-Albalate 2013).

## Materials and methods

Material of *P.depressus* from Moldova, used in this study, was recently received by RJ from Dr. Ilya Osipov (USA) and identified, based on the keys provided in Pollock (1991) and Háva and Zahradník (2021). The beetles from Serbia were observed personally by DC, while VT and RF collected data for *P.abieticola* from Lithuania. Single observations uploaded in the iNaturalist.org data base (www.inaturalist.org/observations/174250744 and www.inaturalist.org/observations/189178097), as well as in the Macrogamta.lt online photogallery (www.macrogamta.lt/lt/fotografija/eglinis-p%C5%ABzravabalis-pytho-abieticola-24153) were a stimulus for RJ to contact DC, VT and RF and, finally, to prepare this manuscript. All specimens from Moldova mentioned below are currently deposited in the first author’s collection (RJC), while material from Lithuania is deposited in the Kaunas Tadas Ivanauskas Museum of Zoology collection (KZM).

The Catalogue of Palearctic Coleoptera (Pollock 2008, Pollock and Iwan 2020) and the Fauna Europaea database (Vazquez-Albalate 2013) were accepted as the basis for the distributional checklist of genus *Pytho* of the Palearctic presented below. In addition, data overlooked in these sources, as well as records from recent literature and the new ones, were also given.

## Taxon treatments

### Pytho abieticola

J. R. Sahlberg, 1875

F437917B-3317-52BB-A34F-6892AFD26FFF

#### Materials

- **Type status:** Other material. **Occurrence:** recordedBy: Vytautas Tamutis; occurrenceID: 3E7B8E53-ED03-51F6-8A37-4E15212ACEC1; **Location:** country: Lithuania; municipality: Ignalina District Municipality; locality: Ažvinčių sengirės gamtinis rezervatas [Azhvinschiai Primeval Forest Nature Reserve]; verbatimCoordinates: N55.434908 E26.065562; **Identification:** identifiedBy: Vytautas Tamutis; **Event:** samplingProtocol: flight interception trap; eventDate: 22.05.2020.–12.06.2020.; habitat: *Piceaabies* dominated forest; **Record Level:** type: 2 specimens; collectionID: KZM
- **Type status:** Other material. **Occurrence:** recordedBy: Romas Ferenca; occurrenceID: ABBC3F00-763A-5E03-8AE3-BB141AD637F3; **Location:** country: Lithuania; municipality: Kaišiadorys District Municipality; locality: Šešuva Reserve; verbatimCoordinates: N54.934772 E24.252475; **Identification:** identifiedBy: Romas Ferenca; **Event:** eventDate: 15.03.2021.; fieldNotes: under bark of dead pine; **Record Level:** type: 1 specimen; collectionID: KZM; collectionCode: KZM IC-56300
- **Type status:** Other material. **Occurrence:** recordedBy: Vytautas Tamutis; occurrenceID: 7DE992D5-00D2-5115-8F6D-0BC44B0BA2ED; **Location:** country: Lithuania; municipality: Alytus District Municipality; locality: Punios šilas [Punia forest]; verbatimCoordinates: N54.545196 E24.022972; **Identification:** identifiedBy: Vytautas Tamutis; **Event:** samplingProtocol: flight interception trap; eventDate: 19.05.2023.–05.06.2023.; habitat: *Piceaabies* dominated forest; **Record Level:** type: 1 specimen; collectionID: KZM; source: https://www.inaturalist.org/observations/189178097
- **Type status:** Other material. **Occurrence:** recordedBy: Vytautas Tamutis; occurrenceID: 1FB36BFD-6EDA-557A-80DA-AC08132861DE; **Location:** country: Lithuania; municipality: Alytus District Municipality; locality: Punios šilas [Punia forest]; verbatimCoordinates: N54.545055 E24.030973; **Identification:** identifiedBy: Vytautas Tamutis; **Event:** samplingProtocol: flight interception trap; eventDate: 19.05.2023.–05.06.2023.; habitat: *Piceaabies* dominated forest; **Record Level:** type: 1 specimen; collectionID: KZM

#### Description

**Figure 1.**
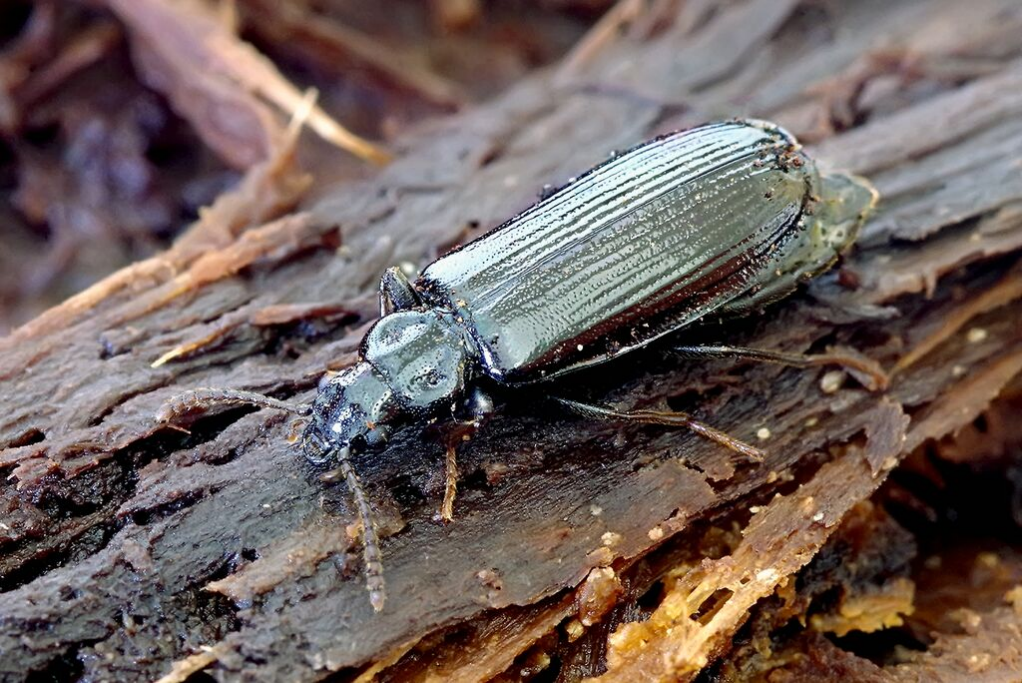
Specimen of *Pythoabieticola* J. R. Sahlberg, 1875 from the Šešuva Reserve, Kaišiadorys District Municipality, Lithuania (photo Romas Ferenca).

Small to medium beetles with moderately flattened bodies; body length 5.6–10.6 mm (9.5 mm in specimen from the Šešuva Reserve, others not measured). Head, pronotum and elytra black, legs and antennae brown-black (Fig. 1). Ratio of pronotal width-length less than 1.5 in most specimens, mesosternum impunctate or with few punctures.

#### Distribution

**Figure 2.**
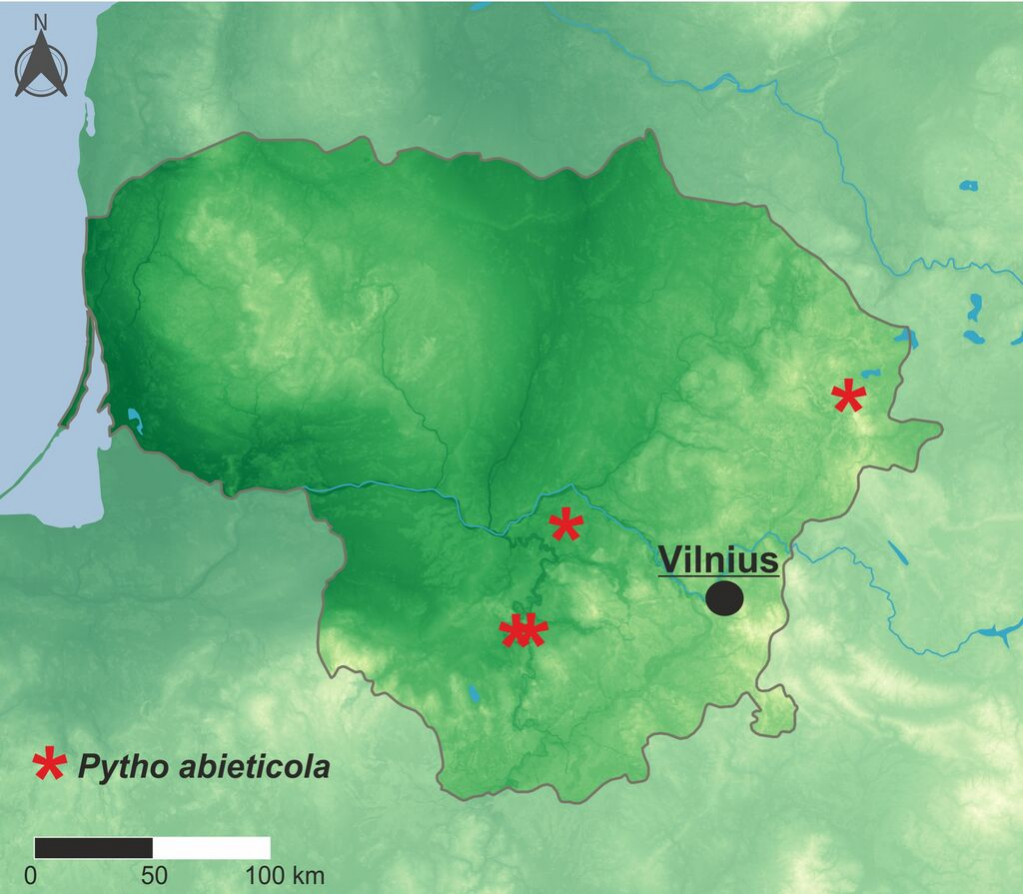
Distribution of *Pythoabieticola* J. R. Sahlberg, 1875 in Lithuania.

Palearctic taxon. Similar to other *Pytho* species, *P.abieticola* prefers regions with lower annual temperatures; in Central and Western Europe, it is restricted mainly to mountainous areas. Till now, it has been recorded from 12 countries (for details see distributional checklist below), including the first records from Lithuania presented here (Fig. 2).

#### Ecology

**Figure 3.**
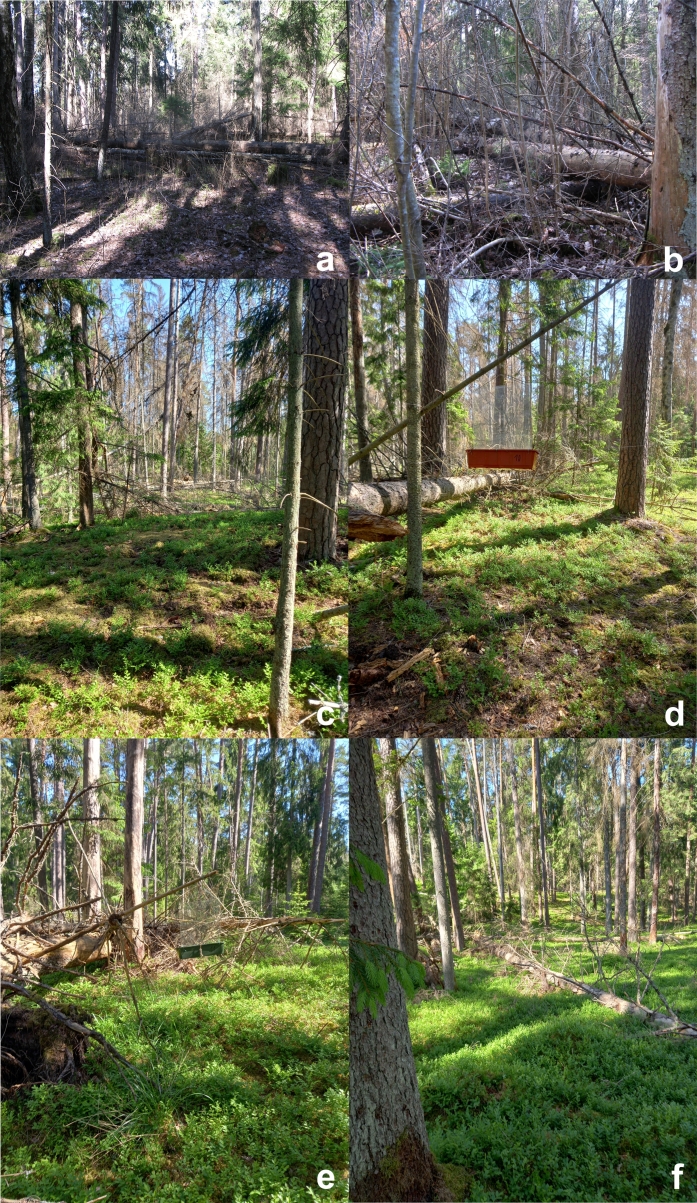
Habitats of *Pythoabieticola* J. R. Sahlberg, 1875 in Lithuania: **a, b** Šešuva Reserve (N54.934772 E24.252475), Kaišiadorys District Municipality; **c, d** Punios šilas (N54.545196 E24.022972), Alytus District Municipality; **e, f** Punios šilas (N54.545055, E24.030973), Alytus District Municipality. Note the flight interception trap in photos d-e (photos a & b - Romas Ferenca, c-f - Vytautas Tamutis).

Numerous data suggest that *P.abieticola* is exclusively associated with spruce forests (*Picea* spp.), often restricted to primeval forest areas (Saalas 1917, Saalas 1923, Burakowski et al. 1987, Pollock 1991, Horák 2017) where it usually inhabits swampy sites (Saalas 1917, Saalas 1923). It has been also reported from pine (*Pinus* spp.) and fir (*Abies* spp.) trees (Koch 1989); one of our findings suggests that the species can also live under the bark of fir trees. Fallen, freshly dead trees lying horizontally and with no direct contact with the ground and characterised by small or medium diameter (6-25 cm) are preferred by females to lay their eggs. Larvae then develop under the bark (Saalas 1917, Saalas 1923, Burakowski et al. 1987). The trees used for development are previously colonised by different bark beetles, especially *Pityogeneschalcographus* (Linnaeus, 1760), *Hylastes* spp. and *Polygraphus* spp. (Curculionidae, Scolytinae). Habitats of *Pythoabieticola* in Lithuania are shown in Fig. 3.

There are no regular studies focused on feeding preferences or feeding behaviour in *P.abieticola*. Some literature data suggest that larvae of this species are zoophagous (Koch 1989), which can be supported by observations of their cannibalistic behaviour observed under "artificial" conditions by Sahlberg (1875) and Saalas (1923), while other authors suggest decaying cambial-phloem layer and/or fungi as the main type of food (Chittaro et al. 2023).

#### Conservation

*Pythoabieticola* is listed in category I on the list of primeval forest relict species of Central Europe, which includes taxa restricted to a few remnants of natural forests (Eckelt et al. 2017). Globally, the species is considered as "least concern" (category LC) (Pettersson et al. 2010), but this situation should be changed soon (Chittaro et al. 2023) as it is threatened or even regionally extinct in all European countries where it has been noted. Cálix et al. (2018) placed *P.abieticola* on the IUCN Red List of European Saproxylic Beetles as a species of "near threatened" (NT) category. In Austria (Jäch et al. 1994) and in Germany (Schmidl et al. 2021), it is recognised as "regionally extinct" (RE), in the Czech Republic (Horák 2017) and in Norway (Ødegaard et al. 2021) as "critically endangered" (CR), in Poland (Pawłowski et al. 2002) as "endangered" (EN), in Sweden (SLU Artdatabanken 2020) as "vulnerable" (VU) and in Finland (Malmberg et al. 2019) as "near threatened" (NT). Actually, some populations of this species are protected in national parks and/or nature reserves, for example, in Switzerland (Chittaro et al. 2023), Poland (Burakowski 1962, Kubisz and Tsinkevich 2001, Kubisz 2004), Czech Republic (Hošek 2008), China (Painter et al. 2007), Russia (Painter et al. 2007), Finland (Finnish Biodiversity Information Facility 2023a) and Lithuania (this paper).

#### Biology

Based on Burakowski et al. (1987), larval development takes at least three years and larvae of various sizes can be found throughout the year (Saalas 1923). The pupal stage takes about two weeks (Sahlberg 1875) and can be found between the second half of July and the first half of September (Burakowski et al. 1987). As in all *Pytho* species, pupal cells under bark are used by adults as shelters to overwinter. In April and May, the adults start to be active and reproduce.

### Pytho depressus

(Linnaeus, 1767)

B4CAA9F0-14CE-5379-BB59-7D8B2E474FD1

#### Materials

- **Type status:** Other material. **Occurrence:** recordedBy: col. local collector; occurrenceID: CA3E8475-ED52-5CE4-9FB1-5CAD1DAA4799; **Location:** country: Moldova; municipality: Municipality of Chișinău; locality: Chișinău: Rose Valley Park; verbatimCoordinates: N47.002119 E28.852038; **Identification:** identifiedBy: Radomir Jaskuła; **Event:** eventDate: 18.12.2019.; **Record Level:** type: 3 specimens; collectionID: RJC; collectionCode: RJC/P005; RJC/P006; RJC/P007
- **Type status:** Other material. **Occurrence:** recordedBy: Denis Ćoso; occurrenceID: 77F8868F-715A-58B5-9300-DCBA72D33244; **Location:** country: Serbia; stateProvince: City of Belgrade District; municipality: Municipality of Voždovac; locality: Beli Potok; verbatimCoordinates: N44.705656 E20.522934; **Identification:** identifiedBy: Enrico Ruzzier; **Event:** eventTime: 28.01.2018.; fieldNotes: under bark of dead pine; **Record Level:** type: 1 specimen (photographed by Denis Ćoso)
- **Type status:** Other material. **Occurrence:** recordedBy: Denis Ćoso; occurrenceID: 965E1100-F89E-586E-99FE-DBE34EB2D519; **Location:** country: Serbia; stateProvince: City of Belgrade District; municipality: Municipality of Voždovac; locality: Beli Potok; verbatimCoordinates: N44.705656 E20.522934; **Identification:** identifiedBy: Denis Ćoso; **Event:** eventDate: 06.02.2018.; fieldNotes: under bark of dead pine; **Record Level:** type: 1 specimen (photographed by Denis Ćoso)
- **Type status:** Other material. **Occurrence:** recordedBy: Denis Ćoso; occurrenceID: 83F31E9D-B988-504B-8775-3F6260A2B3A2; **Location:** country: Serbia; stateProvince: City of Belgrade District; municipality: Municipality of Voždovac; locality: Beli Potok; verbatimCoordinates: N44.705656 E20.522934; **Identification:** identifiedBy: Denis Ćoso; **Event:** eventDate: 02.03.2018.; fieldNotes: under bark of dead pine; **Record Level:** type: 2 specimens (photographed by Denis Ćoso)
- **Type status:** Other material. **Occurrence:** recordedBy: Denis Ćoso; occurrenceID: 84AF98F7-B5C7-5E3B-B981-A9A6E7A56646; **Location:** country: Serbia; stateProvince: City of Belgrade District; municipality: Municipality of Voždovac; locality: Beli Potok; verbatimCoordinates: N44.706434 E20.523825; **Identification:** identifiedBy: Denis Ćoso; **Event:** eventDate: 02.12.2018.; fieldNotes: under bark of dead pine; **Record Level:** type: 1 specimen (observed by Denis Ćoso)
- **Type status:** Other material. **Occurrence:** recordedBy: Denis Ćoso; occurrenceID: 37383745-A505-561A-BC3B-5F519E817230; **Location:** country: Serbia; stateProvince: City of Belgrade District; municipality: Municipality of Voždovac; locality: Beli Potok; verbatimCoordinates: N44.706434 E20.523825; **Identification:** identifiedBy: Denis Ćoso; **Event:** eventDate: 27.01.2019.; fieldNotes: under bark of dead pine; **Record Level:** type: 1 specimen (observed by Denis Ćoso)
- **Type status:** Other material. **Occurrence:** recordedBy: Denis Ćoso; occurrenceID: C89F44FA-9A39-5385-95D7-D0AA3152E65D; **Location:** country: Serbia; stateProvince: City of Belgrade District; municipality: Municipality of Voždovac; locality: Beli Potok; verbatimCoordinates: N44.706434 E20.523825; **Identification:** identifiedBy: Denis Ćoso; **Event:** eventDate: 09.02.2020.; fieldNotes: under bark of dead pine; **Record Level:** type: 1 specimen (observed by Denis Ćoso)
- **Type status:** Other material. **Occurrence:** recordedBy: Denis Ćoso; occurrenceID: 354BF2F3-2CF2-5BE6-A02E-93A8677AA082; **Location:** country: Serbia; stateProvince: City of Belgrade District; municipality: Municipality of Voždovac; locality: Beli Potok; verbatimCoordinates: N44.706516 E20.524496; **Identification:** identifiedBy: Denis Ćoso; **Event:** eventDate: 07.01.2021.; fieldNotes: under bark of dead pine; **Record Level:** type: 1 specimen (observed by Denis Ćoso); source: www.inaturalist.org/observations/174250744

#### Description

Relatively small beetles with strongly flattened bodies, body length (measured from anterior margin of clypeus to the end of elytra) of three studied specimens from Moldova (Serbian individuals photographed in the field were not measured): 7.86 mm, 10.64 mm and 12.13 mm. Pronotum widest in the middle, with sides not constricted anteriorly, clypeus 3.2 times wider than long. In examined specimens head, pronotum and elytra were brown-bluish, legs brown-black and antennae brown-yellow, with yellowish pale body.

#### Distribution

**Figure 4.**
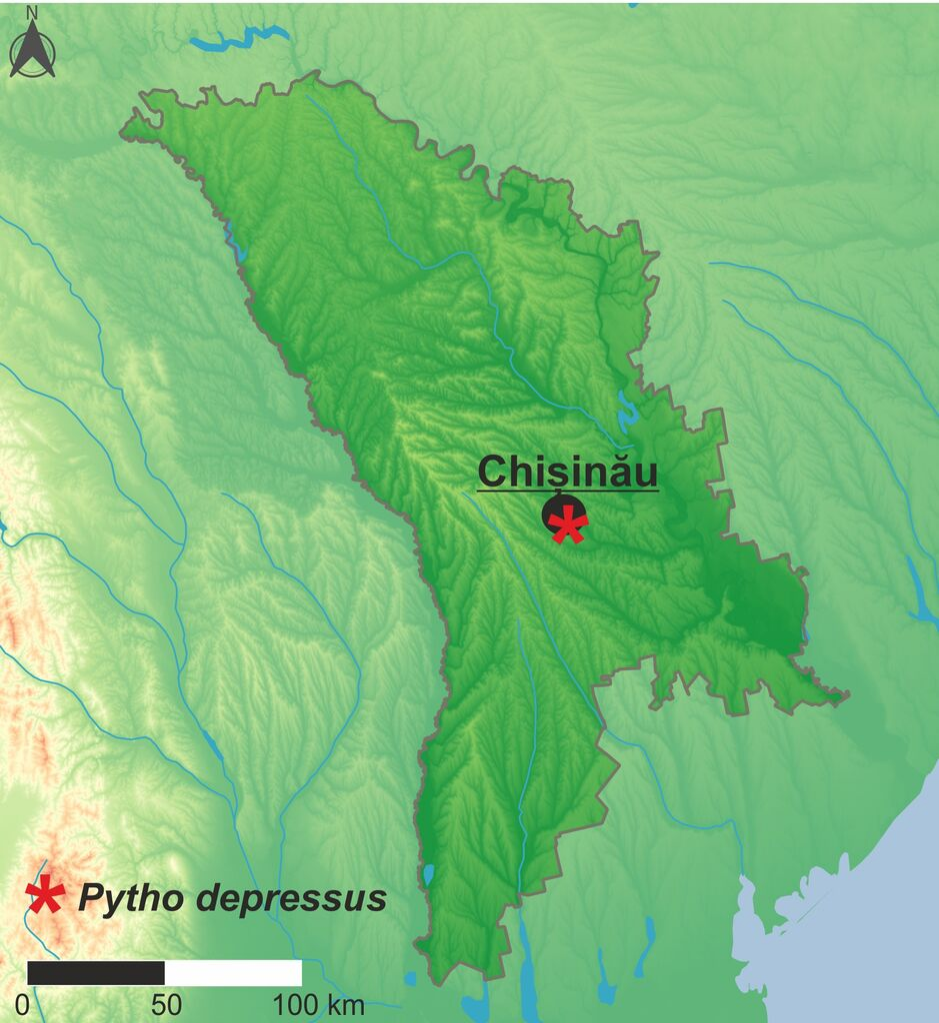
Distribution of *Pythodepresus* (Linnaeus, 1767) in Moldova.

**Figure 5.**
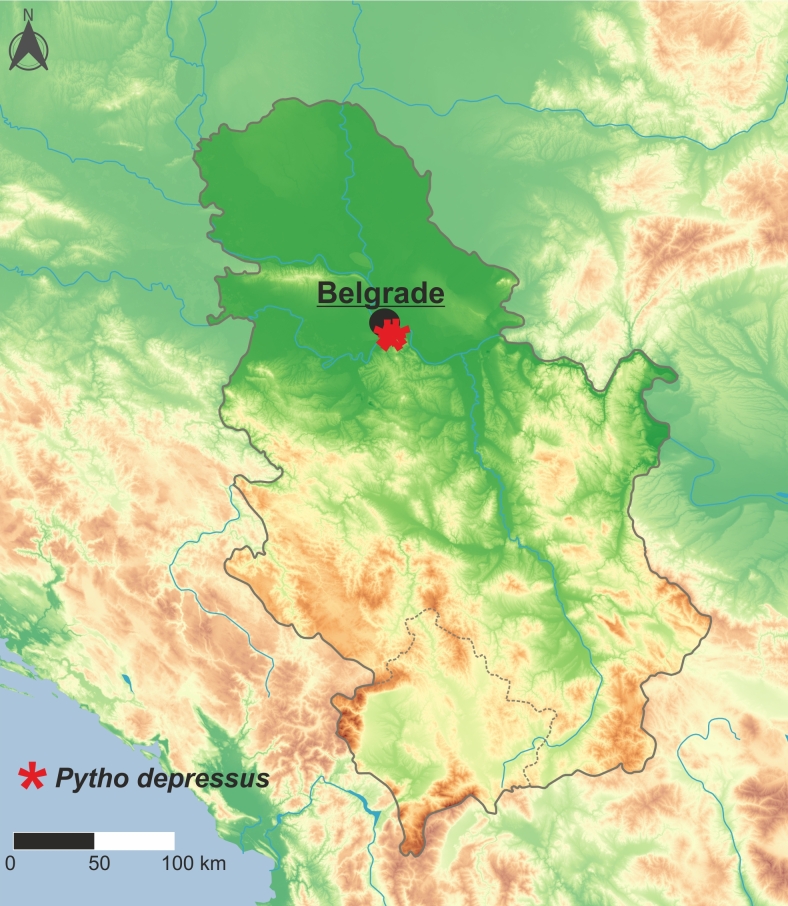
Distribution of *Pythodepressus* (Linnaeus, 1767) in Serbia.

Palaearctic taxon. Like most members of the genus, *P.depressus* prefers cold boreal regions and are more sporadic outside such areas. Until now, it has been recorded from 32 countries, including the first records from Moldova (Fig. 4) and Serbia (Fig. 5) presented in this paper, making it the most widely distributed species amongst all Palaearctic *Pytho* (for details, see distributional checklist below). The occurrence of this species in central Italy (Dias et al. 2021), from where its the southern-most record in Europe is known, should be confirmed by new data as it is possible that the material was mislabelled as this locality is placed outside the species' distribution (Vázquez-Albalate 1993).

#### Ecology

Different literature sources (e.g. Pollock (1991), Miessen (1994), Siitonen and Saaristo (2000), Smith and Sears (2012), Kapla et al. (2021), Chittaro et al. (2023)) show that *P.depressus* occurs in forested areas from lowlands to mountains (up to 2200 m a.s.l.). Large host tree trunks are preferred, but occasionally wood pieces up to 6 cm in diameter are also accepted. Dead pines (*Pinus* spp.), both fallen and standing trees, are selected by adults as places for development, but it has furthermore been noted on other dead conifers, such as spruce (*Picea* spp.), fir (*Abies* spp.) and larch (*Larix* spp.) and, occasionally, even on deciduous tree species including birch (*Betula* spp.), willow (*Salix* spp.) and poplar (*Populus* spp.). Moreover, Pollock (1991) mentioned that rarely *P.depressus* lives in still fresh trees with the bark still intact and without fungal activity. In eastern Fennoscandia, Ananyev et al. (2022) noted this species from a burnt area where its larvae were quite abundant under the bark of coniferous trees in fire-affected spruce forest.

Literature data suggest that a variety of food accepted by larvae of this species – from (mainly) rotting phloem and pulp, sawdust from the food of other insect larvae, their excrement and microorganisms living amongst the mentioned materials to (rarely) larvae of other saproxylic beetles (Saalas 1917, Larsson 1945, Palm 1951, Kaszab 1969, Burakowski 1962, Anderson and Nilssen 1978, Smith and Sears 2012). The physiological anti-freezing adaptations observed in this species shows that it can survive freezing down to -27^o^C (Zachariassen 1979, Lundheim and Zachariassen 1993).

Based on observations from Russian Karelia, Martikainen and Koponen (2001) suggest that *Meteoruscorax* Marshall, 1898 (Hymenoptera, Braconidae) is a parasite of *P.depressus* larvae. In Ukraine, a tarsonemid mite *Tarsonemusmetacinops* Kaliszewski, 1993 (Acari, Tarsonemidae) was recorded under the elytrae of *P.depressus* (Khaustov and Magowski 2003).

#### Conservation

Cálix et al. (2018) listed *Pythodepressus* in the IUCN Red List of European Saproxylic Beetles as least concern (LC category), as it is the most widely distributed taxon amongst all Palaearctic species classified in the genus. On the other hand, it is necessary to note that the population trends of *P.depressus* are poorly studied. Moreover, in some regions or even countries, the species is rare or sporadic and often occurs in isolated populations (e.g. Benedikt 2011, Chittaro and Sanchez 2016, Háva and Zahradník 2021, Kapla et al. 2021, Thomaes et al. 2022) which potentially can be threatened in the future. Unsustainable forestry, especially logging and removal of large dead trees for economical, health and safety reasons, is recognised as one of the main threats for this saproxylic species even if it is known as a habitat generalist (Laaksonen et al. 2008). Some populations of this species are protected in national parks and/or nature reserves, for example, in Switzerland (Chittaro et al. 2023), Poland (e.g. Burakowski et al. 1987, Szafraniec 1997, Kubisz and Tsinkevich 2001, [Bibr B10629705][Bibr B10629740], Szafraniec (1997), Kubisz and Tsinkevich (2001), Kubisz (2004), Marczak 2020, Buchholz et al. 2021 ,Marczak et al. 2023), Belarus (Lukin 2010), Bulgaria (Kovács et al. 2011), China (Painter et al. 2007), Russia (e.g. Painter et al. 2007, Alekseev 2014, Egorov et al. 2020, Ananyev et al. 2022, Ruchin et al. 2022), Finland (Finnish Biodiversity Information Facility 2023b) and the United Kingdom (Jaskuła - unpublished).

#### Biology

*Pythodepressus*, like all members of the genus, is a saproxylic species and its life cycle takes at least two years (Burakowski 1976). Shortly after copulation, males die while females lay eggs in small clusters of several pieces under the bark of host trees in May-June. The sperm ultrastructure in this species was described by Dias et al. (2021). Embryonic development takes about two weeks. Larvae are elongated (reaching 22–30 mm) with body yellowish pale and live under loose bark (Burakowski 1976, Iablokoff-Khnzorian 1985, Pollock 1991). Pupation usually takes place in August or September (Burakowski 1976). Adults overwinter in pupal chambers; they can be characterised by a small body size (7.5 to 13.7 mm long) and a dorsoventrally flattened body well adapted for activity and movements in the cambial layer, a microhabitat also shared by larvae.

## Discussion

The checklist of the Palaearctic species of *Pytho* includes only six species that make up 60% of the world fauna (Pollock and Iwan 2020, Háva and Zahradník 2021). The distribution data for five of them have been summarised by Pollock (2008) and more recently by Pollock and Iwan (2020) in the Catalogue of Palaearctic Coleoptera. The authors included the following number of countries in species ranges of particular taxa: *P.abieticola* – 9, *P.depressus* – 26, *P.jezoensis* – 1, *P.kolwensis* – 5, *P.nivalis* – 2. Unfortunately, they overlooked distributional data for three of these species including records of *P.abieticola* from China (Painter et al. 2007), *P.depressus* from Belgium (Boosten 1971, Mayné 1975, Leroux 1976, Lhost 1976, Lhost 1993, Miessen 1994, Thomaes et al. 2022), Bulgaria (Kovács et al. 2011), South Korea (Kim et al. 2004) and China (Painter et al. 2007), as well as for *P.kolwensis* from China (Painter et al. 2007). In addition, the data for *P.depressus* from Belgium and Bulgaria are missing also in the Fauna Europaea data base (Vazquez-Albalate 2013), in which the distribution of Pythidae species was summarised for the European continent; the reason for this most probably is a consequence of acceptance of the Catalogue of Palaearctic Coleoptera as the main source of distributional data. The actual updated distributional checklist of the genus *Pytho* in the Palearctic realm looks as follows:

1) *Pythoabieticola* J. R. Sahlberg, 1875: Austria, Czech Republic, Finland, Germany, Norway, Poland, Russia, Slovakia, Sweden (Pollock and Iwan 2020), China (Painter et al. 2007), Switzerland (Chittaro et al. 2023), Lithuania (this paper);

2) *Pythodepressus* Linnaeus, 1767: Austria, Bosnia and Hercegovina, Belarus, Russia, Czech Republic, Denmark, Estonia, Finland, France, Great Britain, Germany, Georgia, Hungary, Italy, Kazakhstan, Latvia, Lithuania, the Netherlands, Norway, Poland, Romania, Slovakia, Spain, Sweden, Switzerland, Ukraine (Pollock and Iwan 2020), Bulgaria (Kovács et al. 2011), South Korea (Kim et al. 2004), Slovenia (Kapla et al. 2021), China (Painter et al. 2007, Háva and Zahradník 2021), Belgium (Mayné 1975, Boosten 1971, Lhost 1976, Lhost 1993, Leroux 1976, Miessen 1994, Thomaes et al. 2022Boosten 1971, Leroux 1976,), Moldova, Serbia (this paper);

3) *Pythojezoensis* Kono, 1936: Japan (Pollock and Iwan 2020);

4) *Pythokolwensis* C. R. Sahlberg, 1833: Estonia, Finland, Russia, Poland, Sweden (Pollock and Iwan 2020), China (Painter et al. 2007);

5) *Pythonivalis* Lewis, 1888: Russia, Japan (Pollock and Iwan 2020);

6) *Pythosichuanensis* Háva et Zahradník, 2021: China (Háva and Zahradník 2021).

Previous studies on the genus *Pytho* and the new findings presented herein allow the conclusion that, amongst all mentioned *Pytho* species, one is endemic to Japan (*P.jozoensis*; Yoshitomi and Hayashi 2015, Pollock and Iwan 2020) and one to China (*P.sichuanensis*; Háva and Zahradník 2021). *Pythonivalis* is actually known from two countries, but future intensive studies probably will reveal its presence in the Korean Peninsula as this region is located between Russian Far East and Japan where the species is known to occur (Iablokoff-Khnzorian 1985, Egorov 1992, Yoshitomi and Hayashi 2015, Pollock and Iwan 2020); this area is characterised by proper habitats for this beetle. *Pythokolwensis*, which is a very rare and globally endangered taxon (Cálix et al. 2018), is known only from six countries (Painter et al. 2007, Pollock and Iwan 2020), but it disappeared in numerous localities during recent decades because of human activity, particularly unsustainable forestry (Saalas 1923, Jansson and Palm 1936, Siitonen and Saaristo 2000). In the EU countries, this species and its habitas are protected by a law under the Natura 2000 network (Heikkinen et al. 2021). *Pythoabieticola* has been recorded from 12 countries, in most of them being known as rare (e.g. Painter et al. 2007, Kubisz et al. 2014, Horák 2017, Chittaro et al. 2023). *Pythodepressus* is characterised by the widest species range amongst all species in the genus (occurrence in 33 countries) and the number of known localities for this beetle is also the highest. However, the data presented in this paper, including its first records for the fauna of Moldova and Serbia, clearly suggest that, even for such well known Pythidae species, still there are some significant gaps in its distribution. An important fact is that some of our new country records from both mentioned countries were available thanks to citizen science including the iNaturalist database and the online photogallery. This clearly shows the importance of citizen scientists in studies focused on distribution and diversity of insects, especially in case of rarely investigated groups, which can be easily overlooked, for example, because of lack of specialists in the region. As was previously shown for flat bark beetles (Cucujidae) (Jaskuła et al. 2021a, Jaskuła et al. 2021b, Jaskuła et al. 2022), a beetle family similar to Pythidae because of comparable body size and hidden life style, citizen science can be a crucial tool in future studies documenting the distribution of *Pytho* species.

## Supplementary Material

XML Treatment for Pytho abieticola

XML Treatment for Pytho depressus
